# Surgical Management of Ebstein’s Anomaly in Elderly Patients: A Report of Two Cases

**DOI:** 10.7759/cureus.88877

**Published:** 2025-07-28

**Authors:** Yoshihiko Onishi, Takashi Miyamoto, Kagami Miyaji

**Affiliations:** 1 Cardiovascular Surgery, Kitasato University School of Medicine, Sagamihara, JPN

**Keywords:** adult congenital heart disease (achd), appropriate timing for surgery, cardiac surgery, ebstein's anomaly, elderly patient

## Abstract

Ebstein’s anomaly (EA) is a rare congenital defect of the tricuspid valve (TV), typically characterized by downward displacement of the septal and posterior leaflets into the right ventricle, resulting in tricuspid regurgitation (TR), right heart enlargement, and heart failure. While surgical outcomes for EA have improved significantly in pediatric and young adult populations, data on surgical intervention in elderly patients remain limited. Elderly patients often present with comorbidities and diminished physiological reserve, which complicate both surgical decision-making and perioperative management. We present two elderly patients with EA who underwent TV surgery.

Case 1 involved a 73-year-old man with a long-standing history of severe TR, cardiomegaly, alcoholic cirrhosis, and chronic kidney disease. Despite preserved biventricular systolic function on echocardiography, signs of right ventricular dysfunction were present. The patient underwent tricuspid valve replacement (TVR) due to concerns about tissue fragility and prolonged bypass time. Although initially stable postoperatively, he developed aspiration pneumonia followed by septic shock and ultimately died from multiorgan failure on postoperative day 73. Case 2 involved a 71-year-old woman with preserved cardiac function and mild frailty. She underwent tricuspid valvuloplasty using the cone technique. Despite temporary postoperative circulatory instability requiring mechanical circulatory support, she recovered well and was discharged on postoperative day 80.

These cases highlight the importance of individualized surgical planning in elderly EA patients. While echocardiographic indices may suggest preserved ventricular function, they may not fully reflect clinical reserve, particularly in long-standing disease. Earlier surgical intervention, prior to the onset of irreversible right ventricular remodeling or systemic decline, may improve outcomes even in elderly patients. Evaluation of frailty, comorbidities, and right ventricular function should guide the decision-making process. Our experience suggests that timely and tailored surgical strategies can lead to acceptable outcomes in selected elderly patients with EA.

## Introduction

Ebstein's anomaly (EA) is a rare congenital malformation of the tricuspid valve (TV), accounting for less than 1% of all congenital heart diseases, with an estimated incidence of approximately 2.5 per 20,000 live births [1]. The hallmark anatomical feature is the downward (apical) displacement of the septal and posterior leaflets of the TV into the right ventricle. This displacement causes a portion of the right ventricle to become functionally incorporated into the right atrium - a phenomenon referred to as atrialization. As a result, EA is associated with varying degrees of tricuspid regurgitation (TR), right-sided chamber enlargement, and heart failure symptoms. Conventional surgical criteria for EA include the presence of heart failure symptoms (e.g., New York Heart Association (NYHA) class III-IV), severe TR, progressive right atrial or ventricular enlargement, and impaired exercise capacity [2-4]. Surgical outcomes in children and younger adults have improved considerably over the past decades due to refinements in techniques such as the cone reconstruction, which reconfigures the malformed TV into a more functional structure. However, evidence regarding surgical intervention in elderly patients with EA remains limited. A multicenter analysis by Attenhofer Jost et al. reported that fewer than 5% of untreated EA patients survive beyond age 50 [2], and most surgical reports to date have focused on younger populations. The elderly often present with comorbidities, such as pulmonary disease, renal impairment, or frailty, that may complicate surgery and prolong recovery [5,6]. This clinical complexity underscores the limitations of applying standard surgical criteria directly to older patients. Some studies suggest that earlier surgical intervention, initiated before symptom onset or substantial cardiomegaly, may improve outcomes, even among elderly adults [7,8]. Still, evidence in this population remains sparse. In this report, we present two rare cases of EA in elderly patients who underwent surgical correction. We examine the perioperative challenges, postoperative outcomes, and broader implications for surgical decision-making in aging EA populations. This experience prompts reconsideration of current surgical indications and highlights the importance of individualized strategies in elderly patients with EA.

## Case presentation

Case 1

A 73-year-old man had been diagnosed with EA and atrial fibrillation (AF) at the age of 38 through echocardiographic evaluation for palpitations. He also had a history of Buerger’s disease but was not hospitalized at that time. He had been under regular follow-up at our hospital. Although surgical treatment had been recommended for some time, the patient declined, and follow-up was conducted with medical therapy alone. Over the years, his TR progressed to a severe grade, accompanied by worsening cardiomegaly. He had been prescribed diuretics - furosemide, spironolactone, and tolvaptan - along with perindopril erbumine for cardiac protective agent; however, his heart failure symptoms, including reduced exercise tolerance, gradually deteriorated. He was admitted to our hospital for acute exacerbation of heart failure. Given the limited effectiveness of conservative treatment, he opted for surgical intervention. His past medical history included alcoholic cirrhosis, chronic kidney disease, Buerger’s disease, and idiopathic epilepsy.

**Figure 1:**
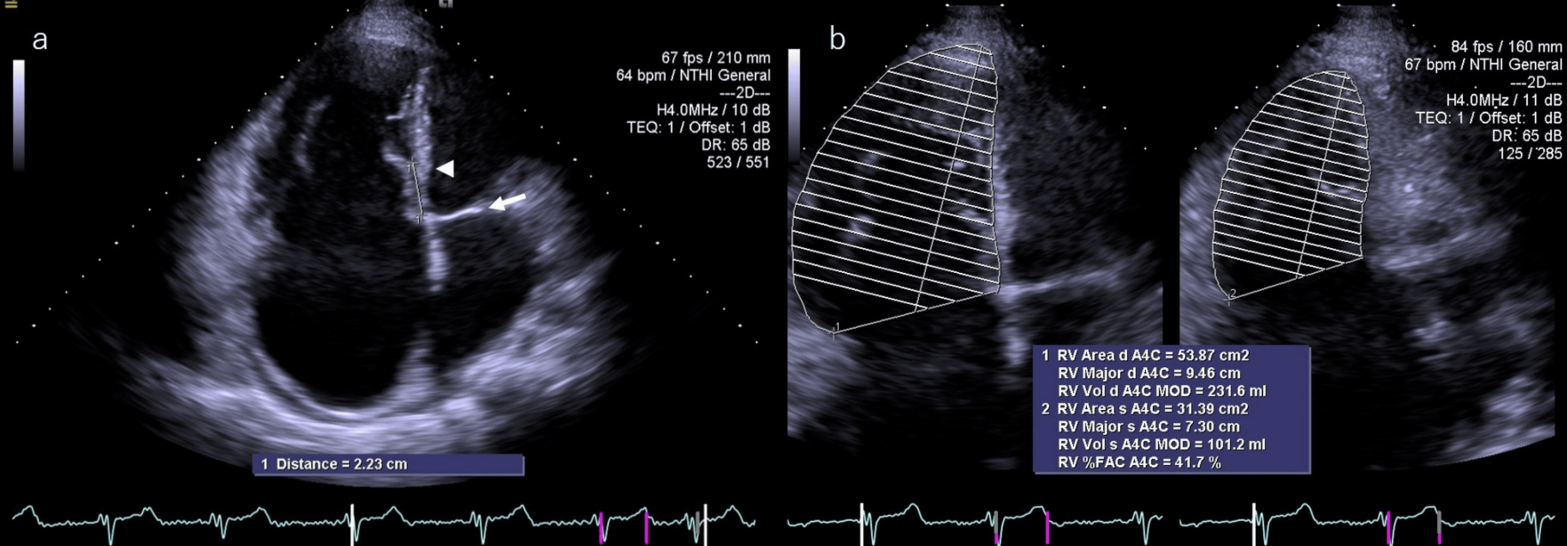
Preoperative transthoracic echocardiography in Case 1 (a) The septal leaflet of the tricuspid valve was displaced approximately 22 mm apically from the anterior mitral leaflet. The arrow marks the position of the anterior mitral leaflet, and the arrowhead indicates the location of the tricuspid septal leaflet. (b) Preoperative assessment of right ventricular function. RV Area d; Right ventricular end-diastolic area; A4C: apical four-chamber view; RV Major d: major axis diameter in diastole; RV Vol d: right ventricular end-diastolic volume; MOD: modified Simpson’s method; RV Area s: right ventricular end-systolic area; RV Major s: right ventricular major axis diameter in systole; RV Vol s: right ventricular end-systolic volume; RV %FAC: right ventricular % fractional area change

Preoperatively, the patient's liver cirrhosis was classified as Child-Pugh class A, and his seizures were well controlled. On admission, physical examination revealed bilateral wheezes on chest auscultation, normal first and second heart sounds (S1 and S2), and a Levine grade 2/6 systolic murmur at the third left sternal border. Jugular venous distention and bilateral lower extremity edema were present. The patient's level of frailty was evaluated as 5 on the Clinical Frailty Scale (CFS), indicating mild to moderate frailty. Laboratory findings showed a platelet count of 96,000/μL, aspartate aminotransferase (AST) 35 U/L, alanine aminotransferase (ALT) 19 U/L, creatinine 1.48 mg/dL, and brain natriuretic peptide (BNP) 369.9 pg/mL. Chest X-ray showed moderate cardiomegaly (cardiothoracic ratio, CTR = 62.3%) and bilateral dull costophrenic angles with mild pulmonary congestion. Electrocardiography (ECG) revealed AF with incomplete right bundle branch block. Transthoracic echocardiography (TTE) revealed apical displacement of the septal cusp of the TV by approximately 22 mm from the anterior cusp of the mitral valve (MV), consistent with atrialization of the right ventricle. The anterior cusp of the TV appeared elongated. Severe TR (grade IV) and right atrial enlargement were noted. The tricuspid regurgitation pressure gradient (TRPG) was 17 mmHg. Biventricular systolic function was preserved, with a left ventricular ejection fraction (LVEF) of 54% and right ventricular fractional area change (RVFAC) of 41.7%. Right ventricular function was further assessed by tricuspid annular plane systolic excursion (TAPSE), which was 16.3 mm, and inferior vena cava (IVC) diameter measurements of 29 mm on inspiration and 23 mm on expiration. The distal right ventricular outflow tract (dRV-OT) was notably enlarged at 53 mm, and the left atrial diameter (LAD) was also enlarged at 51 mm. The left ventricle remained non-dilated with a left ventricular diastolic/systolic dimension (LVDd/Ds) of 41/30 mm. S′ velocity was not assessed (Figure 1).

The patient’s predicted operative mortality risk, based on the European System for Cardiac Operative Risk Evaluation II (EuroSCORE II), was 8.35%. Although biventricular function was preserved on TTE, progressive right ventricular enlargement and the presence of comorbidities such as liver cirrhosis led us to opt for tricuspid valve replacement (TVR) rather than valvuloplasty, in order to minimize perfusion and cardiac arrest times as much as possible. Surgical intervention was performed via median sternotomy.

Cardiopulmonary bypass (CPB) was established using an aortic cannulation and bicaval venous cannulation. Plastering of the septal cusp of the TV between the 1 and 5 o’clock positions to the interventricular septum was noted. We performed TVR at the anatomical tricuspid annulus with a 29-mm Carpentier-Edwards Perimount Magna mitral valve (Edwards Lifesciences, Irvine, CA, USA), plication of the atrialized right ventricle with autologous pericardial strips, and left atrial appendage resection using the Echelon Flex 60-mm Endopath stapler (Ethicon, Inc., a Johnson & Johnson, Cincinnati, OH, USA). Transesophageal echocardiography (TEE) confirmed no major paravalvular leak. The patient was weaned from CPB with inhaled nitric oxide (iNO). The total operative time was 687 minutes, with CPB and aortic cross-clamp times of 167 and 116 minutes, respectively. Due to marked tissue fragility and intraoperative thrombocytopenia leading to increased bleeding tendency, achieving hemostasis was challenging and contributed to the prolonged operative time. The patient was admitted to the intensive care unit (ICU) postoperatively and was initially hemodynamically stabilized. The iNO was discontinued, and he was extubated on postoperative day 6. However, shortly after ICU discharge, he developed fever, hypoxemia, and hypotension. Aspiration pneumonia was suspected, and empirical antimicrobial therapy was modified: levofloxacin was initiated following initial administration of piperacillin/tazobactam. Norepinephrine infusion was commenced for circulatory support. Subsequent deterioration suggested septic shock, and meropenem and vancomycin were introduced, both adjusted to dialysis-appropriate dosages. Blood cultures later revealed *Stenotrophomonas maltophilia*, prompting a switch to intravenous sulbactam/ampicillin and minocycline, also titrated for renal replacement therapy. Sedation was maintained with midazolam, and dopamine hydrochloride (DOA) infusion was added for circulatory support. Continuous hemodiafiltration (CHDF) was initiated on postoperative day 23. Vasopressin was added to support blood pressure and enable fluid removal. Recombinant thrombomodulin was administered in response to laboratory findings consistent with disseminated intravascular coagulation. Despite these interventions, the patient developed progressive multi-organ failure involving both renal and hepatic systems. High-dose intravenous methylprednisolone (1,000 mg/day for three days) was administered for worsening respiratory status attributed to interstitial pneumonia suspect. Ultimately, the patient's condition deteriorated, and the patient passed away on postoperative day 73.

Case 2

**Figure 2:**
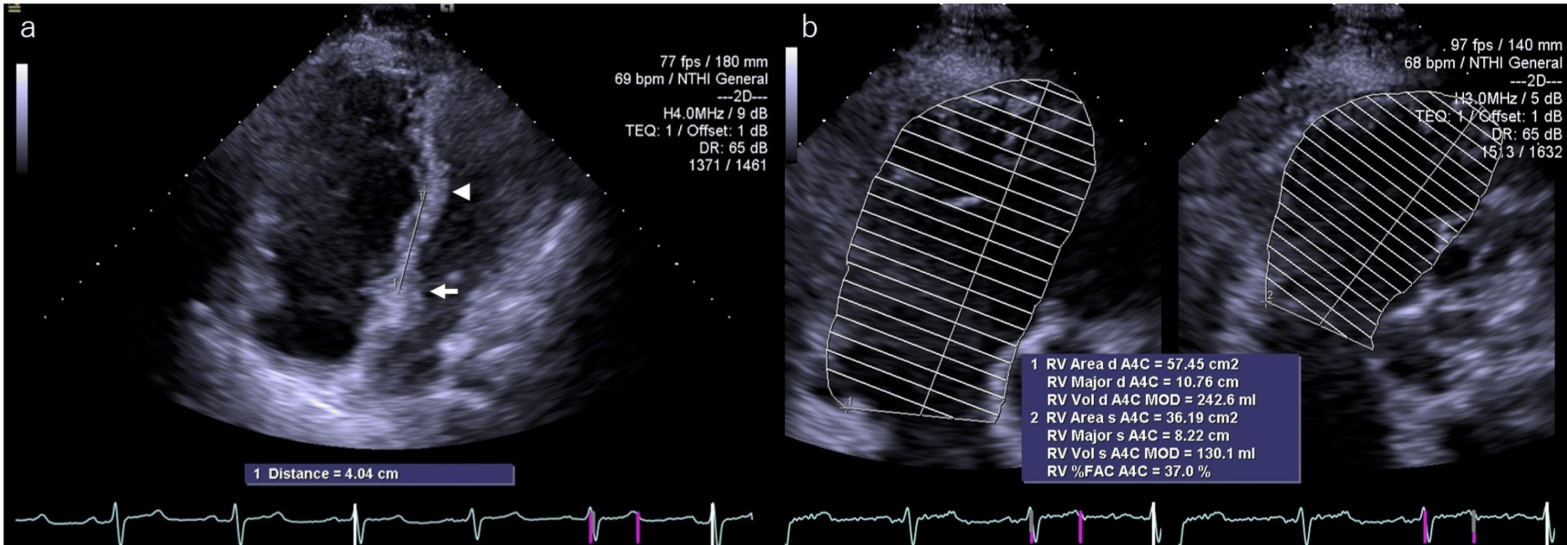
Preoperative transthoracic echocardiography in Case 2 (a) The septal leaflet of the tricuspid valve was displaced approximately 40 mm apically from the anterior mitral leaflet. The arrow marks the position of the anterior mitral leaflet, and the arrowhead indicates the location of the tricuspid septal leaflet. (b) Preoperative assessment of right ventricular function. RV Area d; Right ventricular end-diastolic area; A4C: apical four-chamber view; RV Major d: major axis diameter in diastole; RV Vol d: right ventricular end-diastolic volume; MOD: modified Simpson’s method; RV Area s: right ventricular end-systolic area; RV Major s: right ventricular major axis diameter in systole; RV Vol s: right ventricular end-systolic volume; RV %FAC: right ventricular % fractional area change

A 71-year-old woman had been diagnosed with EA during a school health screening in childhood and had since been followed at a local clinic for EA and TR. She was referred to our hospital for evaluation of progressive heart failure symptoms, classified as NYHA class III. She had been under follow-up at our secondary prevention center for seven years prior to the surgery. Her past medical history included cerebral infarction, ovarian cysts, hypertension, and dyslipidemia. The patient was receiving Camucia LD (a fixed-dose combination of candesartan cilexetil and amlodipine besylate), vonoprazan fumarate, clopidogrel, rosuvastatin calcium, bisoprolol fumarate, and furosemide. On physical examination, heart sounds were normal, with a Levine grade 2/6 systolic murmur at the third left sternal border. No jugular venous distension was observed, but bilateral lower leg edema was noted. The patient was considered to have a CFS score of 3 to 4, indicating mild frailty or vulnerability. Laboratory findings showed a platelet count of 376,000/μL, AST 27 U/L, ALT 25 U/L, creatinine 0.71 mg/dL, and BNP 184.4 pg/mL. Chest X-ray revealed moderate cardiomegaly (CTR = 66.2%) with clear costophrenic angles and no pulmonary congestion. ECG showed sinus rhythm with incomplete right bundle branch block. TTE demonstrated apical displacement of the tricuspid septal leaflet by approximately 40 mm from the anterior mitral leaflet, accompanied by atrialization of the right ventricle. Enlargement of both the right atrium and right ventricle was observed, dRV-OT 19 mm, with plastering noted along the atrialized portion. The anterior leaflet of the TV appeared elongated. Severe TR (grades III-IV) was present. The TRPG was 28.9 mmHg. Right ventricular systolic function was preserved, with an RVFAC of 37.0% and a TAPSE of 27.2 mm. The IVC measured 15.2 mm during inspiration and 10.2 mm during expiration. Left ventricular function was well maintained, with an LVEF of 67.3%, LVDd/Ds of 28.4/15.6 mm, and no left ventricular enlargement. The LAD was 35.2 mm, indicating no significant left-sided chamber dilation. S′ velocity was not assessed (Figure 2).

The patient’s predicted operative mortality risk, based on EuroSCORE II, was 3.94%. Given the preserved cardiac function and absence of progressive biventricular dilation, our initial strategy was to attempt tricuspid valvuloplasty (TVP), with TVR reserved for cases in which regurgitation could not be sufficiently controlled. She underwent surgical correction via median sternotomy with CPB established using an aortic cannulation and bicaval venous cannulation. Significant plastering of the septal cusp of the TV between the 3 and 6 o’clock positions to the interventricular septum was noted. We performed the TVP with the cone technique, annuloplasty with a 28-mm MC^3^ ring (Edwards Lifesciences), plication of the atrialized right ventricle, and direct closure of a 3-mm atrial septal defect. The cone procedure included individually detaching the TV cusp, then reconstructing extracorporeally and reimplanting at the anatomical tricuspid annulus. Following leaflet reattachment, a cleft between the posterior and septal cusps was identified, and we performed primary closure. TEE showed no residual TR or intracardiac shunting. She was weaned from CPB with ventricular pacing with iNO. The total operative time was 644 minutes, with CPB and aortic cross-clamp times of 220 and 138 minutes, respectively. The procedure required excision, re-suturing, and reattachment of the valve leaflets, which prolonged the operative time. Additionally, marked tissue fragility necessitated extended efforts for hemostasis, further contributing to the length of the surgery. Postoperatively, the patient was admitted to the ICU. On postoperative day 1, due to unstable hemodynamics, she required mechanical circulatory support (MCS) with veno-arterial extracorporeal membrane oxygenation (VA-ECMO) and intra-aortic balloon pumping (IABP). On the same day, right-sided pleural effusion drainage was performed. Because vasodilatory shock was also present, high-dose norepinephrine was required. Given the clinical suspicion of sepsis, broad-spectrum antimicrobial therapy was initiated. As the patient’s hemodynamic status gradually stabilized with MCS and multidisciplinary therapeutic interventions, she was successfully weaned off VA-ECMO on postoperative day 7 and IABP on postoperative day 9, followed by extubation on postoperative day 14. After medical therapy for heart failure and rehabilitation, she was discharged home on postoperative day 80.

A summary of the patients’ preoperative evaluations, including echocardiography, ECG, chest X-ray, and laboratory findings, is shown in Table 1.

**Table 1:** Preoperative examination results of patients TTE: transthoracic echocardiography; RVFAC: right ventricular fractional area change; TAPSE: tricuspid annular plane systolic excursion; TV: tricuspid valve; MV: mitral valve; TRPG: tricuspid regurgitation pressure gradient; LVDd/Ds: left ventricular diastolic/systolic dimension; RV-OT distal: distal right ventricular outflow tract; LAD: left atrial diameter; IVC: inferior vena cava; CTR: cardiothoracic ratio; IRBBB: incomplete right bundle branch block

	Reference range	Case 1	Case 2
TTE			
Ejection fraction (%)	≥50% preserved, 40-49% mildly reduced, <40 reduced	54	67
RVFAC (%)	≥35%	41.7	37.0
TAPSE (mm)	≥17 mm	16.3	27
Apical displacement of TV from MV (mm)	<20 mm	22.3	40.4
TRPG (mmHg)	<30 mmHg	17	28.9
LVDd/Ds (mm)	40-55 mm/30-40 mm	41/30	28/15
RV-OT distal diameter (mm)	17-27 mm	53	19
LAD (mm)	≤40 mm	51	35
IVC diameter (mm)	≤21 mm with respiratory variation >50% on inspiration	29/23	15/10
Electrocardiography		Atrial fibrillation with IRBBB	Sinus rhythm with IRBBB
Chest X-ray			
CTR (%)	≤50%	62.3	66.2
Costophrenic angles	Clear	Bilateral dull	Clear
Congestion	-	Mild	None
Laboratory findings			
Platelet (/μL)	150,000-350,000/μL	96,000	376,000
Aspartate aminotransferase (AST) (U/L)	≤30 U/L	35	27
Alanine aminotransferase (ALT) (U/L)	≤30 U/L	19	25
Creatinine (mg/dL)	Male: 0.65～1.07 mg/dL; Female: 0.46～0.79 mg/dL	1.48	0.71
Brain natriuretic peptide (BNP) (pg/mL)	18.4 pg/mL	369.9	184.4

## Discussion

EA is characterized by morphological abnormalities of the TV and the right ventricle, and is one of the rarest congenital heart diseases, occurring in approximately 2.5 per 20,000 live births [1]. The history of EA is extremely variable; some reports describe the symptoms of heart failure in neonates, and others describe an asymptomatic course into adulthood. Thus, there is a wide age range of patients to be treated [4]. However, the natural course of most of the EA patients is not good, with less than 5% of patients generally surviving beyond the age of 50 years without surgical intervention [2,5]. In recent years, thanks to the development of surgical techniques and perioperative management, the operative mortality rate, which used to be high, has significantly improved to less than 4% [2,3].

Presently, TTE is most commonly used for diagnosis because it is simple and noninvasive, and likewise for follow-up examinations. In EA patients, the septal and posterior cusps of the TV are displaced downward into the apex. In neonates and children, EA is defined as a distance >8 mm/m^2^ body surface area (BSA) between the anterior cusp of the MV and the location of the displaced TV or >20 mm in adults [6]. Malcoaptation of the TV due to displacement causes TR, and its severity depends on the degree of displacement. The displaced valve divides the right ventricle into an atrialized right ventricle that functions as the right atrium and a functional right ventricle that forms the right ventricular outflow tract. The American Society of Echocardiography (ASE) guidelines recommend TAPSE, S′ velocity, and RVFAC as core indicators of right ventricular function. While 3D right ventricular ejection fraction and right ventricular strain provide more precise assessments, their applicability is limited in settings without the appropriate device [7]. Although conventional echocardiography can be used to diagnose morphological abnormalities, nowadays, cardiac magnetic resonance imaging (CMRI) is considered to be the superior modality for functional evaluation [8]. The most recent guidelines for surgical indications for EA patients are the 2020 European Society of Cardiology (ESC) Guidelines for adult congenital heart diseases (ACHDs), which state that surgery is indicated in cases of severe TR, symptomatic conditions, and decreased exercise tolerance [9]. Several reports advocate deciding on surgical intervention based on whether the patients have symptoms or not [10,11]. However, timely surgery is necessary in patients of all ages before significant cardiomegaly and cardiac function decline occur [12]. In cases of patients with good ventricular function, the threshold for surgery can be lowered [10].

Surgical techniques for EA include TVP, such as cone and Carpentier surgery, and TVR with an artificial valve. There is no clear indication at this time for the choice of technique. Patients with advanced tethering and insufficient right ventricular volume due to the TV tissue in the right ventricular outflow tract may require valve replacement instead of inadequate valvuloplasty [11]. Some reports suggest that patients with valve replacement have a better prognosis than those with inadequate valvuloplasty, especially if the patient is older than 50 years old. In such patients, we should lower the threshold for valve replacement [4,5,13]. The percentage of freedom from reoperation following initial surgery for EA was 86%, 74%, 62%, and 46% after 5, 10, 15, and 20 years, respectively [14]. Some reports show that plication of the right ventricle reduced reoperation [5] and no significant difference in the reoperation rate at 12 years between valvuloplasty and replacement [15]. It is important that we choose carefully, according to each patient's individual status, whether to perform valvuloplasty or valve replacement.

However, there are a few reports of such in patients older than 65 years. In ACHD patients, potential organ damage has been reported, such as the presence of vascular lesions at a young age [16,17] and concomitant renal dysfunction due to congestion caused by chronic heart disease [18,19]. In both cases, biventricular systolic function appeared preserved based on LVEF and RVFAC measurements. However, in Case 1, indices such as TAPSE and RV outflow tract diameter were outside the normal range, whereas these remained within normal limits in Case 2. Although RVFAC in Case 1 suggested some preserved right ventricular function, the actual clinical course revealed marked RV dysfunction. Postoperatively, this contributed to persistent low cardiac output and progression of multiorgan failure. It is conceivable that the delayed timing of surgery, partly influenced by patient refusal, may have contributed to the poor outcome, and that earlier intervention could have prevented mortality even in the presence of major complications. In Case 2, ventricular function assessed by TTE was preserved; however, reliance solely on echocardiographic parameters may have limitations in evaluating right ventricular performance. Additional imaging modalities such as CMRI, known for their accuracy in assessing RV function, should be considered when available.

In general, elderly patients with well-preserved cardiac function, manageable comorbidities, and low frailty can often undergo cardiac surgery with acceptable risk and outcomes. However, patients with ACHD, who have been exposed to abnormal hemodynamics since birth, likely possess reduced organ reserve compared to age-matched non-ACHD individuals. In Case 1, due to the presence of significant comorbidities and preoperative heart failure symptoms, TVR was planned to minimize CPB and cardiac arrest times. In Case 2, valve repair was chosen to preserve the native valve, given the absence of major comorbidities and preserved ventricular function. Although repair was associated with slightly prolonged perfusion and arrest times compared to replacement, the outcome regarding regurgitation control was satisfactory. Nonetheless, a replacement-first strategy might have led to a better clinical course given the need to shorten perfusion time.

Our clinical experience suggests that, in elderly patients with EA, the presence of RV enlargement, such as RVOT dilation, or signs of functional decline, including reduced RVFAC, TAPSE, or S′ velocity, may indicate that cardiac and systemic organ reserve is already compromised or approaching deterioration. In such cases, we advocate for earlier surgical intervention, specifically in the presence of TR of grade 4 or higher, or grade 3 TR accompanied by impaired RV function, irrespective of symptom status. Although this approach may diverge from conventional guideline recommendations, the potential to prevent further deterioration of RV function and systemic capacity may contribute to more favorable postoperative outcomes.

## Conclusions

In elderly patients with EA, standard surgical indications may not adequately capture the clinical complexity and reduced organ reserve commonly seen in this population. The presence of heart failure symptoms in these patients often indicates advanced disease and diminished systemic capacity. Therefore, earlier surgical intervention, before the onset of severe symptoms or significant right ventricular dysfunction, should be considered. An individualized approach that accounts for functional parameters such as RVFAC, TAPSE, and RVOT diameter, rather than symptom status alone, may help improve postoperative outcomes and long-term prognosis in this unique and vulnerable patient group.
